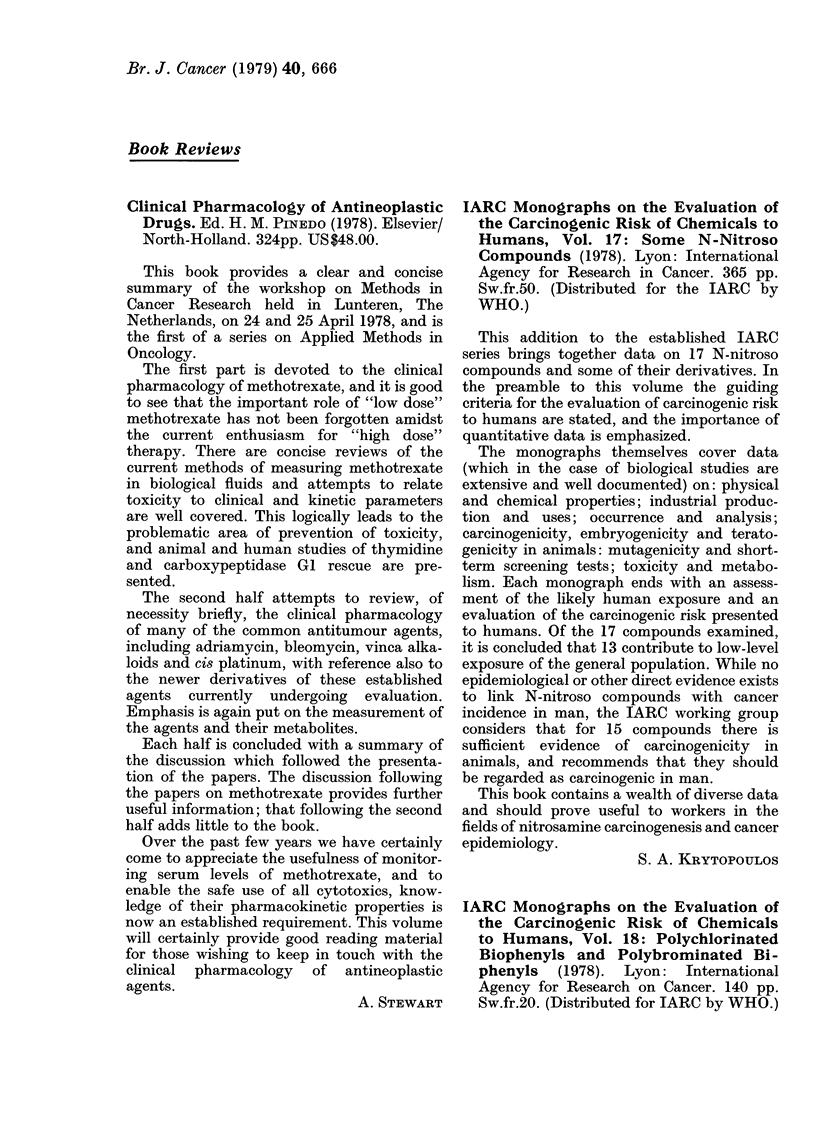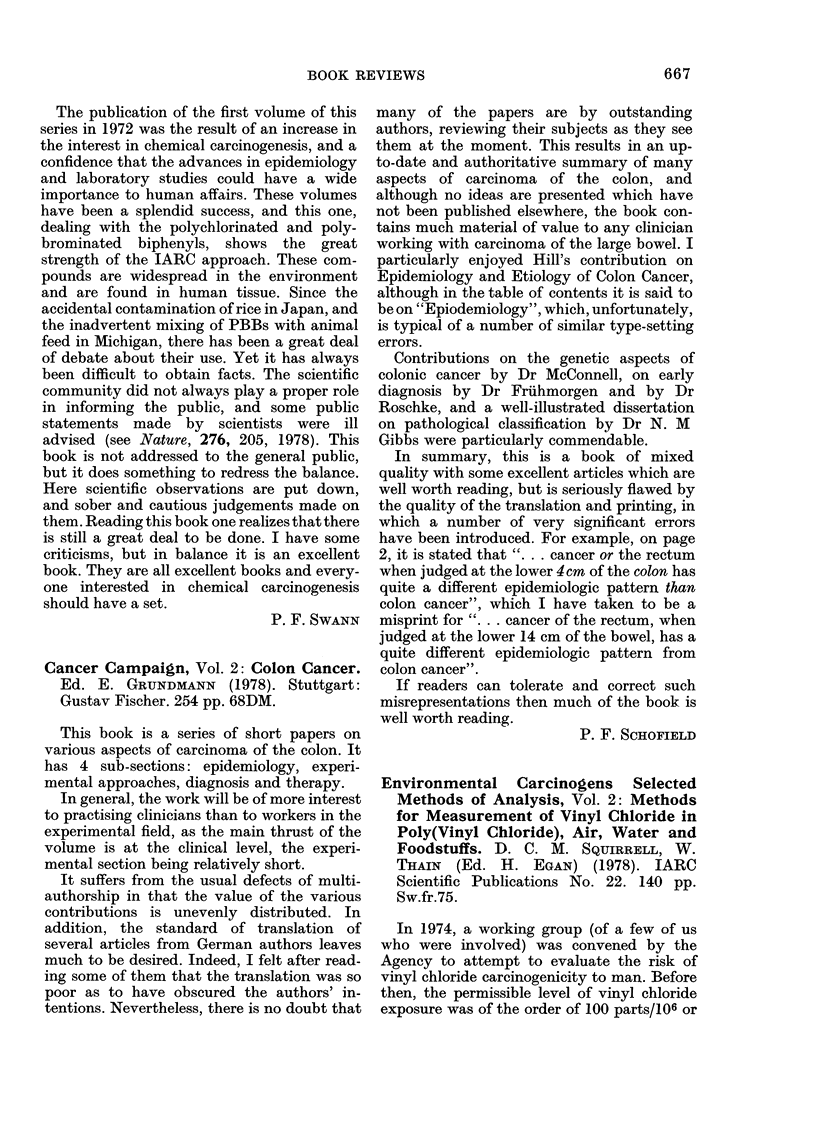# IARC Monographs on the Evaluation of the Carcinogenic Risk of Chemicals to Humans, Vol. 18: Polychlorinated Biophenyls and Polybrominated Biphenyls (1978)

**Published:** 1979-10

**Authors:** P. F. Swann


**IARC Monographs on the Evaluation of the Carcinogenic Risk of Chemicals to Humans, Vol. 18: Polychlorinated Biophenyls and Polybrominated Biphenyls** (1978). Lyon: International Agency for Research on Cancer. 140 pp. Sw.fr.20. (Distributed for IARC by WHO.)

The publication of the first volume of this series in 1972 was the result of an increase in the interest in chemical carcinogenesis, and a confidence that the advances in epidemiology and laboratory studies could have a wide importance to human affairs. These volumes have been a splendid success, and this one, dealing with the polychlorinated and polybrominated biphenyls, shows the great strength of the IARC approach. These compounds are widespread in the environment and are found in human tissue. Since the accidental contamination of rice in Japan, and the inadvertent mixing of PBBs with animal feed in Michigan, there has been a great deal of debate about their use. Yet it has always been difficult to obtain facts. The scientific community did not always play a proper role in informing the public, and some public statements made by scientists were ill advised (see *Nature*, **276**, 205, 1978). This book is not addressed to the general public, but it does something to redress the balance. Here scientific observations are put down, and sober and cautious judgements made on them. Reading this book one realizes that there is still a great deal to be done. I have some criticisms, but in balance it is an excellent book. They are all excellent books and everyone interested in chemical carcinogenesis should have a set.

P. F. SWANN